# The Expanding Toolbox of *In Vivo* Bioluminescent Imaging

**DOI:** 10.3389/fonc.2016.00150

**Published:** 2016-06-23

**Authors:** Tingting Xu, Dan Close, Winode Handagama, Enolia Marr, Gary Sayler, Steven Ripp

**Affiliations:** ^1^The Center for Environmental Biotechnology, The University of Tennessee, Knoxville, TN, USA; ^2^490 BioTech, Inc., Knoxville, TN, USA; ^3^The Department of Biology, Maryville College, Maryville, TN, USA

**Keywords:** bioluminescence, optical imaging, *in vivo* imaging, luciferase, luciferin

## Abstract

*In vivo* bioluminescent imaging (BLI) permits the visualization of engineered bioluminescence from living cells and tissues to provide a unique perspective toward the understanding of biological processes as they occur within the framework of an authentic *in vivo* environment. The toolbox of *in vivo* BLI includes an inventory of luciferase compounds capable of generating bioluminescent light signals along with sophisticated and powerful instrumentation designed to detect and quantify these light signals non-invasively as they emit from the living subject. The information acquired reveals the dynamics of a wide range of biological functions that play key roles in the physiological and pathological control of disease and its therapeutic management. This mini review provides an overview of the tools and applications central to the evolution of *in vivo* BLI as a core technology in the preclinical imaging disciplines.

## Introduction

**Figure 1:**
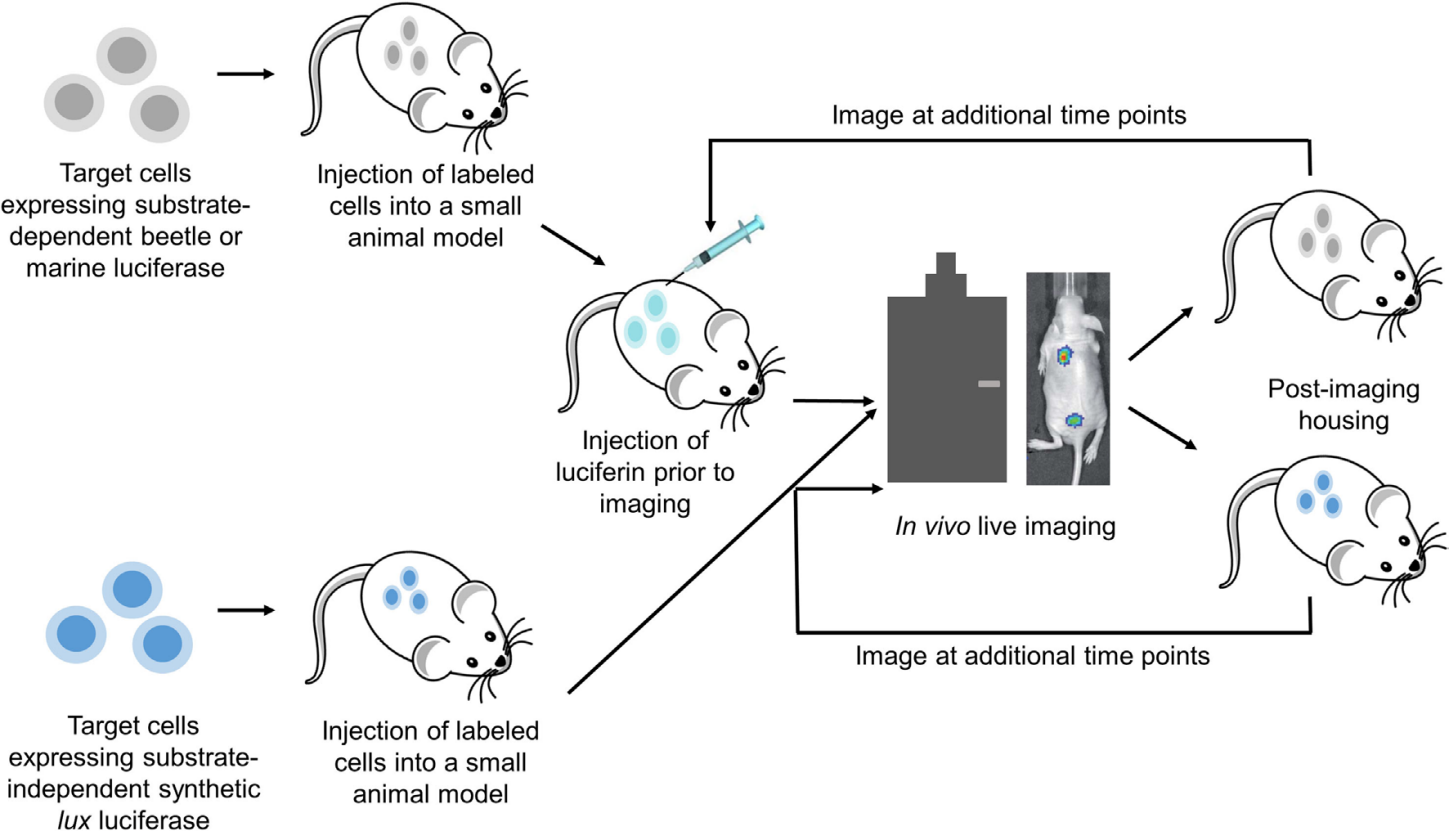
***In vivo* BLI uses advanced camera imaging systems to visualize live animal subjects as they express bioluminescence from targeted cells and tissues, thereby allowing fundamental biological processes to be monitored non-invasively**. Advances in the *in vivo* BLI field have created luciferase proteins with expanded wavelength emission profiles, stronger and more stable signal generation, substrate-independent real-time expression, and proximity-based expression characteristics that are providing innovative tools for preclinical diagnostics, drug discovery, and toxicology research.

*In vivo* bioluminescent imaging (BLI) enables the visualization of biological processes as they occur within the living subject. The information obtained is unprecedented in its ability to elucidate biology beyond the boundaries of the conventional *in vitro* assay, where the complex interactions of a living system are all but ignored. *In vivo* BLI uses the luciferase family of proteins to create signature bioluminescent outputs that are then externally captured by advanced cameras (Figure 1). Luciferases operate in tandem with their luciferin substrates to generate light *via* an oxidation decarboxylation reaction that forms an excited state intermediate that releases energy in the form of photons as it returns to its ground state. In nature, bioluminescence is generated by various bacteria, fungi, protozoa, dinoflagellates, and higher-order terrestrial and marine organisms, with the firefly being the most recognized example. Molecular biology has enabled the genes involved in bioluminescent light reactions to be isolated, manipulated, and reapplied toward applications, such as *in vivo* BLI, where bioluminescence as an optical emission signature exhibits certain unique imaging advantages. Among the most critical is a superior signal-to-noise ratio due to cells and tissues emitting virtually no intrinsic bioluminescence, thus effectively eliminating background interference when probing for a bioluminescent signal within the intricate milieu of a living entity. However, detecting bioluminescence at depths beyond a few centimeters inside of a living animal remains challenging because light signals must be obtained and evaluated after passing through host tissue that absorbs, attenuates, and scatters their emissions (1). This has currently constrained *in vivo* BLI to small animal models, such as mice and rats, with service primarily limited to preclinical imaging applications. This mini review provides an overview of the luciferases currently being applied in *in vivo* imaging applications along with the toolbox of approaches that continue to expand the capabilities of *in vivo* BLI.

## Luciferases for BLI Applications

Luciferases applied in *in vivo* BLI include those derived from beetles, bacteria, and various marine species (Table 1), with the firefly luciferase (FLuc) being the most widely used. FLuc requires d-luciferin (a heterocyclic carboxylic acid), ATP, and molecular oxygen for light production. At a pH between 7.5 and 8.5, FLuc catalyzes the reaction between d-luciferin and ATP to form luciferyl-adenylate, which in the presence of oxygen then undergoes an oxidative decarboxylation reaction to form CO_2_, AMP, and oxyluciferin. Initially formed as an excited state intermediate, oxyluciferin quickly returns to its ground state and releases energy in the form of light (2). In addition to FLuc, other beetle luciferases, such as the green (CBG) and red (CBR) click beetle luciferases, emerald luciferase (ELuc), and stable red luciferase (SRL), also utilize d-luciferin as their substrate. However, despite this common substrate, these luciferases emit light of different wavelengths (Table 1).

**Table 1:** **The inventory of luciferases for *in vivo* BLI applications**.

Luciferase	Luciferin substrate	Peak emission (nm) (25°C)	Reference	Examples of *in vivo* BLI applications
**Beetle luciferases**				
*Photinus pyralis* (firefly; FLuc, *ffluc*, or *luc*) and its enhanced variants (effLuc, luc2)	d-luciferin	560	(3–5)	• Detection of cancer cells and evaluation of tumor treatment (3, 5) • Imaging of neural precursor cell migration to glioma tumor (6) • Imaging of T cell migration to tumors (7) • Split luciferase assay to image apoptosis in response to chemotherapy and radiotherapy in a glioma model (8)
Red-shifted firefly luciferase PRE9	d-luciferin	620	(9)	
*Pyrearinus termitilluminans* (click beetle emerald; ELuc)	d-luciferin	538	(10)	
*Pyrophorus plagiophthalamus* (click beetle red; CBR)	d-luciferin	615	(11)	
*Pyrophorus plagiophthalamus* (click beetle green; CBG)	d-luciferin	540	(12)	
*Phrixothrix hirtus* (railroad worm stable luciferase red; SLR)	d-luciferin	630	(13)	
**Bacterial luciferases**				
*Aliivibrio fischeri, Vibrio harveyi*, and *Photorhabdus luminescens lux* for bacterial expression	FMNH_2_ + long-chain aliphatic aldehyde	490	(14, 15)	• Simultaneous imaging of *lux*-labeled bacterial trafficking to FLuc-tagged tumor (14) • Substrate-free imaging of human cells *in vivo* (16) • Substrate-free real-time imaging of bacterial infection of human cells (17)
Synthetic *lux* for mammalian expression	FMNH_2_ + long-chain aliphatic aldehyde (self-supplied by *luxCDEfrp*)	490	(18, 19)	
**Marine luciferases**				
*Renilla reniformis* (RLuc) and its enhanced variants (RLuc8 and RLuc8.6–535)	Coelenterazine	482–535	(20–22)	• RLuc multiplexed with FLuc to monitor tumor regression in response to therapeutic genes delivery by neural precursor cells (6) • VLuc multiplexed with FLuc and RLuc to track delivery of therapeutic genes into brain tumor (23) • BRET assay to detect tumor metastasis (24)
*Gaussia princeps* (GLuc) and its mutants (I90L, 8990, 90115, Monsta, etc.)	Coelenterazine	482–503	(25, 26)	
*Metridia longa* (MLuc7, MLuc164)	Coelenterazine	486–498	(27, 28)	
*Aequorea victoria* (aequorin)	Coelenterazine	470	(29)	
*Vargula hilgendorfii* (VLuc)	Vargulin	462	(30)	
*Cypridina noctiluca* (CLuc)	Cypridina	460	(31)	
*Oplophorus gracilirostris* (NanoLuc)	Furimazine	460	(32)	
*Benthosema pterotum* (BP)	Coelenterazine	475	(7)	

Within the bacterial genera, bioluminescence from *Photobacterium* and *Aliivibrio*/*Vibrio* are typically applied. These systems encode the *lux* gene cassette, which includes the *luxAB* genes encoding a heterodimeric bacterial luciferase and the *luxCDE* genes encoding a fatty acid synthetase/reductase complex that generates a long-chain fatty aldehyde substrate from endogenous intracellular metabolites. Marine bioluminescent bacteria also possess a *luxG/frp* gene that encodes a flavin reductase that recycles reduced flavin mononucleotide (FMNH_2_) for the luciferase reaction. Similar to beetle luciferases, bacterial luciferase generates light in an ATP-dependent manner in the presence of long-chain aldehyde, FMNH_2_, and molecular oxygen (33). Distinctive to bacterial bioluminescence is the ability of cells expressing the full *luxCDABE* gene cassette to produce light autonomously without the need for exogenous luciferin by self-supplying the aldehyde and FMNH_2_ substrates. While bacterial bioluminescence is traditionally employed to label bacterial pathogens for *in vivo* real-time infection tracking due to its prokaryotic origin, the *lux* cassette has been synthetically optimized for autonomous bioluminescent expression in eukaryotic organisms, allowing substrate-free *in vivo* imaging of mammalian cells (18).

The remaining luciferases include those isolated from marine invertebrates. Unlike other luciferases, marine luciferases utilize their luciferin substrate and molecular oxygen to generate light in an ATP-independent fashion. There is also no common luciferin substrate for all marine luciferases. While *Gaussia* (GLuc), *Renilla* (RLuc), and *Metridia* (MLuc) luciferases share the same substrate coelenterazine, *Cypridina* (CLuc) and *Vargula* (VLuc) luciferases catalyze their reactions using cypridina and vargulin, respectively. Some marine luciferases, including GLuc and MLuc, are naturally secreted outside of the cell, thus allowing bioluminescent detection without cell lysis (25, 27).

## The Toolbox of BLI Approaches

### Mutated and Synthetic Luciferase

The high utility and common limitations shared by luciferases has made them especially attractive targets for synthetic modification. One of the first major synthetic luciferase modifications was a polymutated variant of RLuc, which incorporated eight independent single amino acid changes to increase protein stability and improve light output (20). This mutated variant allowed for improved function during serum exposure in small animals, provided a facile means for conjugating luciferase protein to various ligands (34), and has recently been used for conjugation to immunoglobulin G proteins for visualizing antigen–antibody reactions (35).

Perhaps the most valuable synthetic luciferase modifications for *in vivo* BLI have been those that shift the luciferases’ emission signal further into the red spectrum, thereby improving signal penetration through living tissue. To overcome the naturally blue-shifted emission wavelength of RLuc, Loening et al. (21) generated a library of active site mutations and identified multiple variants with emission spectra peaks ranging from 475 to 547 nm. Branchini et al. (36) employed a similar approach with FLuc that shifted its native 557 nm emission peak to 617 nm.

Leveraging the proteomic sequences of known luciferases, Kim and Izumi (37) applied a consensus sequence-driven mutagenesis strategy to identify amino acids common to copepod luciferases and arranged these sequences under the constraints suggested by a statistical coupling analysis (38) to mimic the natural evolutionary constraints of the proteins. Using this strategy, they designed artificial luciferases (ALucs) that retained favorable emission wavelengths in the 515–548 nm range. This strategy enables the synthetic generation of alternative classes of luciferases for the continued expansion of BLI beyond those found in nature.

### Synthetic Luciferin Analogs

For *in vivo* BLI to occur under the majority of luciferase/luciferin combinations, the luciferin substrate must first be injected into the animal and then diffuse to where the luciferase-expressing cells are located. This series of events can be challenging. The biodistribution of luciferin substrates in small animals is not homogenous, individual eukaryotic cells are limited in their ability to freely take up substrate, and the mere presence of the luciferin substrate represents a chemical contaminant that may unknowingly introduce experimental artifacts and/or toxicological side effects. Synthetic luciferins with properties better tuned to the *in vivo* environment are being developed to address some of these problems. Craig et al. (39) created some of the early chemically modified (esterified) d-luciferin analogs designed for improved cellular uptake kinetics and consequent near sixfold increases in bioluminescence output. However, for *in vivo* imaging, the focus has transitioned to red-shifting the emission wavelength for improved tissue penetration. This has resulted in aminoluciferin analogs, such as cyclic aminoluciferins and seleno-d-aminoluciferins, with wavelength emissions around 600 nm (40). Unfortunately, the majority of these analogs yield lower light intensities than conventional luciferin, although the CycLuc1 substrate reported by Evans et al. (41) does demonstrate superior photon flux under non-saturating substrate conditions. Infra-luciferin (λ_max_ = 706 nm), a π-conjugated analog (λ_max_ = 675 nm), and CycLuc10 (λ_max_ = 648 nm) have successfully shifted their wavelengths even further into the far-red regions (42–44). However, maintaining elevated photon yields remains challenging, although the increased efficiency of signal penetration at these longer wavelengths does offer heightened resolution.

### Multiplexed BLI

In multiplexed BLI, the subject is tagged with multiple luciferases that utilize different substrates, which are injected sequentially to trigger each bioluminescent signal to enable simultaneous monitoring of multiple biological processes. Common luciferase combinations include d-luciferin-activated beetle luciferase and coelenterazine-activated marine luciferase. The selectivity and specificity of luciferin substrates ensures minimal cross talk. This approach has been applied to monitor gene expression and promoter activities (45), mesenchymal stem cell differentiation (46), and cell migration and tumor apoptosis (6, 47). A triple BLI system consisting of the FLuc/d-luciferin, GLuc/coelenterazine, and VLuc/vargulin pairs has also been reported for simultaneous monitoring of three distinct biological events in an orthotopic brain tumor model (23). However, using multiple substrates inevitably introduces biases due to differential substrate biodistribution and uptake in animal tissues. Meanwhile, multiple substrate injections can be stressful for the animal and introduce potential operational errors. These drawbacks can be alleviated by utilizing a single substrate to simultaneously initiate multiple luciferases that emit light of separable colors. A common approach is to employ one luciferase with a green emission spectrum and a second luciferase emitting a more red-shifted wavelength. Upon a single-substrate application, both luciferases are activated, and the resulting green and red light signal can be spectrally resolved using appropriate detection systems. Beetle luciferases activated by d-luciferin, including FLuc, CBG, and CBR, are currently the most common reporters used for single-substrate multicolor BLI applications (13, 48–51). However, for *in vivo* applications, this arrangement is still constrained due to increased absorption and attenuation of the shorter wavelength (green) light compared to that of the red-shifted signal in animal tissues, which introduces potential detection biases.

### Split Luciferases

Split luciferases, or luciferase fragments, are unique tools for probing protein–protein interactions. Instead of using the complete enzyme, the luciferase protein is split into a C-terminus fragment and an N-terminus fragment that are not capable of catalyzing the bioluminescent reaction on their own. In split luciferase complementation assays, each luciferase fragment is attached to each partner of the interacting peptides, domains, and/or full proteins. Upon interaction of the proteins of interest, the luciferase fragments are brought to a close proximity to form a complete and functional enzyme that produces bioluminescence when a luciferin substrate is available (52). Luciferase fragment complementation imaging can be designed to directly identify interacting protein pairs (53) and to indirectly report protein–protein interactions induced by various biological processes, such as binding of intracellular messengers (e.g., cyclic AMP and Ca^2+^) (54, 55), protein kinase activities (56, 57), caspase-3-mediated apoptosis (8), and activation and/or inhibition of disease-related cell signaling pathways (58, 59). Multiple luciferases can also be used for multiplexed examination of complex interactions involving multiple protein partners simultaneously in the same subject (12, 60). For improved *in vivo* applications, novel split sites and modifications of the luciferase enzyme are being continuously identified to enhance their characteristics (i.e., decreased basal activity, increased specificity, improved signal-to-noise ratio) (61).

### Caged Luciferin

The caged luciferin reporter system uses a luciferin substrate that has been modified such that it cannot interact with its complementary luciferase to generate bioluminescence until an enzymatic cleavage event occurs (62). Lugal (d-luciferin-*O*-β-galactoside) is one example of a caged luciferin that only actively interacts with FLuc upon removal of its galactoside moiety by β-galactosidase. Thus, the bioluminescent reporter cell remains “dark” even after the addition of the Lugal substrate, with the co-addition of β-galactosidase being required to ultimately initiate light emission. Using this strategy, one cell (the reporter cell) can be designed to express FLuc, while another cell (the activator cell) expresses β-galactosidase. If the two cells are in close proximity, then the β-galactosidase released from the activator cell cleaves the Lugal to initiate light emission from the reporter cell. As the distance between these two cells increases, the intensity of the light response correspondingly decreases. For example, this has enabled *in vivo* bioluminescent visualization of tumor metastasis in mouse models, where β-galactosidase-expressing hematopoietic cells distributed throughout a mouse functionally activated luciferase-expressing breast cancer cells that had metastasized from a tumor implant (63). Due to Lugal being somewhat non-selective under biological conditions, other caged luciferin substrates have been developed that operate under a number of more selective enzymatic reaction schemes (β-lactamase, alkaline phosphatase, nitroreductase) (64, 65).

### Bioluminescence Resonance Energy Transfer

Bioluminescence resonance energy transfer (BRET) pairs together two chromophores such that the emission spectra of one (the bioluminescent donor) activates the excitation spectra of the other (the fluorescent acceptor) (66). In its earliest configuration, it exploited the 482-nm bioluminescent emission of *Renilla* luciferase to activate an enhanced yellow fluorescent protein (EYFP), thereby “switching” the blue-green color of RLuc to a 527-nm yellow emission (67). The switching only occurs if the donor and recipient chromophores are properly oriented and situated in close proximity (≤10 nm apart), which enables BRET’s primary application as an indicator of protein–protein interactions *via* the attachment of the donor chromophore to one protein and the recipient chromophore to the other protein (68). BRET has since evolved to include other bioluminescent/fluorescent pairings, which, for *in vivo* applications, have centered on shifting the emission spectra more toward the red to far-red regions to improve tissue penetration (24, 69–71). BRET has also advanced beyond fluorescent proteins to include organic dye (72) and quantum dot conjugates (73).

### Fluorescence by Unbound Excitation from Luminescence

Fluorescence by unbound excitation from luminescence (FUEL) is similar to BRET in that it uses the emission spectra of a bioluminescent donor to activate the excitation spectra of a fluorescent acceptor. However, whereas BRET requires the donor and acceptor to reside within an approximate 10 nm distance of each other, FUEL can theoretically occur at donor/acceptor distances separated by micrometers to centimeters (74, 75). FUEL takes advantage of the unfocused radiative dissemination of photons by luciferase-bearing entities to activate neighboring fluorescent light sources. In one of its earliest demonstrations, *Escherichia coli* cells expressing bacterial luciferase were placed in one quartz cuvette, while red-emitting quantum dots (QD705, Invitrogen) with overlapping excitation wavelengths were placed in a neighboring cuvette. Photons emitted by *E. coli* were shown to activate red-shifted fluorescence from QD705, with signal intensity being dependent on the distance separating the two cuvettes. Injection of bioluminescent bacteria and QD705 into mice showed similar activation of red fluorescence emission under *in vivo* BLI. FUEL may serve as a unique mechanism to gage coproximity of donors and acceptors, much like BRET, but across larger spans of space, for example, to discern interactions between tissues and organs separated on a mesoscopic scale.

### Bioluminescence Assisted Switching and Fluorescence Imaging

Bioluminescence assisted switching and fluorescence imaging (BASFI) is another spin-off of BRET, wherein a bioluminescent donor activates a reversible photoswitchable fluorescent acceptor protein. Proof of concept for BASFI has been demonstrated using the pairing of the bioluminescent Rluc8 donor with the photoswitchable fluorescent protein DG1 acceptor (76). DG1 normally exists in its excited state, emitting green fluorescence at 450–550 nm, but can be switched to an off-state when exposed to wavelengths around 488 nm. A DG1–Rluc8 fusion construct was transfected into a human embryonic kidney cell, thereby endowing it with a green fluorescent phenotype. Exposing the cell to a 488 nm laser switched DG1 to its off-state, and the cell became “dark.” Addition of a coelenterazine methoxy substrate then activated Rluc8, whose 400 nm emission switched DG1 back to its on-state. In traditional BRET, this on-state is transient and short-lived. In BASFI, this on-state persists for as long as the donor bioluminescence is being provided, thereby enabling the accumulation of signal over time. This allows supply of the activation signal to be decoupled from measurement of the emission signal to potentially reduce background and increase sensitivity. BASFI still requires close association between the donor and acceptor (≤10 nm), so its primary application remains with studying protein–protein interactions.

### Bioluminescent Enzyme-Induced Electron Transfer

The bioluminescent enzyme-induced electron transfer (BioLeT) concept uses luciferin analogs that have been modified to contain moieties of differing electron donating capacities and then using the ensuing electron transfer process as an on/off switch to modulate bioluminescent signal output. In its proof-of-concept format, aminoluciferin substrates were modified to contain benzene moieties of differing highest occupied molecular orbital (HOMO) energy levels (77, 78). Substrates containing benzene moieties with high HOMO energy levels, such as a diaminophenyl moiety, were shown to quench bioluminescence when added to a FLuc reaction, presumably due to the electron transfer process occurring much more rapidly than the light-emitting reaction. Substrates containing benzene moieties with low HOMO energy levels did not quench bioluminescent signal output. A diamino-phenylpropyl-aminoluciferin (DAL) substrate was ultimately developed as a BioLeT probe for the targeting of biological nitric oxide. Upon reaction with nitric oxide, the diaminophenyl moiety is converted into a benzotriazole moiety with a lower HOMO energy level, thus transitioning from minimal to a highly bioluminescent output in the presence of luciferase. The scheme was validated *in vivo* in a transgenic FLuc rat model intraperitoneally injected with DAL substrate followed by injection of a NOC7 compound that spontaneously released nitric oxide under physiological conditions. Nitric oxide accumulation was detected *via* increased bioluminescence emission as the diaminophenyl to benzotriazole conversion occurred within the rat. It is anticipated that the BioLeT process can be designed to target other biomolecules, such as singlet oxygen and metal ions, to assist in the real-time, non-invasive surveillance of a subject’s physiological state.

## Conclusion

The superior signal-to-noise ratio due to the absence of intrinsic bioluminescence background in cells and animal tissues has made BLI an attractive tool for investigating biological processes as they occur in real-time in living animals. The past two decades have witnessed not only a bloom in the discovery and engineering of luciferases with improved expression and performance in mammalian cells but also the emergence and expansion of innovative applications of such luciferase reporters for *in vivo* imaging. Despite *in vivo* BLI currently being constrained to small animal models, it has increasingly become a promising tool in preclinical biomedical research to investigate real-time biological events in complex biological systems, and it is reasonable to expect that *in vivo* BLI will continue to play a crucial role in basic research, drug development, disease diagnosis, therapy management, and many other biomedical research and applications.

## Author Contributions

TX and SR conceived, structured, and edited the mini review article. DC, WH, EM, and GS each wrote individual sections of the mini review article and critically revised it for intellectual content. All authors (TX, DC, WH, EM, GS, and SR) provided final approval of the version of the article submitted for publication, and they agreed to be accountable for all aspects of the work in regard to ensuring that questions pertaining to accuracy and/or integrity of any part of the work are appropriately investigated and resolved.

## Conflict of Interest Statement

Drs. DC, GS, and SR have research-related financial interests in 490 BioTech, Inc. The authors declare that the research was conducted in the absence of any commercial or financial relationships that could be construed as a potential conflict of interest. The reviewers (PW and ZC) and handling editor declared their shared affiliation, and the handling editor states that the process nevertheless met the standards of a fair and objective review.
